# Whole-genome re-sequencing association study on body size traits at 10-weeks of age in Chinese indigenous geese

**DOI:** 10.3389/fvets.2024.1506471

**Published:** 2024-12-16

**Authors:** Guobo Sun, Hongchang Zhao, Xiaohui Mu, Xiaoming Li, Jun Wang, Mengli Zhao, Rongchao Ji, Hailing Lv, Yang Li, Chao Chen, Jia Xie, Wei Zhang, Xiujun Duan, Shanyuan Zhu, Jian Wang

**Affiliations:** ^1^Jiangsu Agri-Animal Husbandry Vocational College, Taizhou, China; ^2^National Waterfowl of Gene Pool, Taizhou, China; ^3^Taizhou Fengda Agriculture and Animal Husbandry Technology Co., Ltd., Taizhou, China; ^4^Jiangsu Liangyu Agriculture and Animal Husbandry Co., Ltd., Suqian, China; ^5^Suqian March Food Co., Ltd., Suqian, China

**Keywords:** body traits, re-sequencing, GWAS, FarmCPU, goose

## Abstract

To investigate the genetic factors underlying marketed body size traits in Chinese local geese, we conducted a comprehensive study involving nine body size traits in 251 samples at 10 weeks of age from five local breeds: Taihu goose (TH), Sichuan goose (SC), Guangfeng goose (GF), Xupu goose (XP), and Youjiang goose (YJ). Genotyping data were obtained through whole-genome re-sequencing, followed by a genome-wide association analysis utilizing the fixed and random model circulating probability unification (FarmCPU) approach. Our findings revealed 88 significant SNPs associated with body size traits, with 16 SNPs surpassing the genome-wide significance threshold (*p* = 3.98E-09) and 72 SNPs exceeding the suggestive significance threshold (*p* = 5E-07). Subsequent gene annotation identified these SNPs to be located within exonic regions of 86 candidate genes, including THADA, ATP5A1, ZNF462, PRDM8, and GH14523. Notably, functional enrichment analysis employing Gene Ontology (GO) and Kyoto Encyclopedia of Genes and Genomes (KEGG) pathways highlighted 37 significantly enriched pathways, among which the “negative regulation of transforming growth factor beta receptor signaling pathway” (GO:0030512) emerged as relevant to goose skeletal development and the phenotypic expression of body size in geese. The identification of these novel SNPs and candidate genes associated with 10-week-old body size traits in geese presents valuable insights for future molecular breeding endeavors and the elucidation of underlying mechanisms governing body size trait formation in goose.

## 1 Introduction

The domestic goose, belonging to herbivorous waterfowl, primarily feeds on green coarse forage and possesses characteristics such as fast growth, tolerance to coarse feed, and low feed consumption. This makes geese suitable for both free-range grazing and intensive factory farming, thus making the goose industry an important component of China’s waterfowl industry. In recent years, with the development of the goose industry at a larger scale, an increasing number of large enterprises have joined commercial goose production and placed significant emphasis on goose breeding work. The growth and development capacity of geese is fundamental to their excellent production performance in meat production and plays a crucial role in the economic benefits of the goose industry. In order to enhance the growth and development of geese and increase their economic efficiency, in addition to providing scientifically and rationally managed feeding practices, studying and selecting for the genetic basis of geese through research and breeding can effectively improve their growth and development capacity. From a breeder’s perspective, utilizing genetic approaches to genetically improve the growth-related phenotypic traits of geese can significantly enhance their productivity and economic efficiency. Body size traits are important indicators that measure the size and growth status of geese, influenced by genetic, environmental, and nutritional factors. Therefore, genetic improvement of body size traits holds promise for enhancing the growth and development performance of domestic geese.

However, at present, there is a clear shortage of molecular markers for efficient breeding, necessitating urgent research on functional gene discovery related to the growth and development traits of geese. Genome-wide Association Study (GWAS) is a critical approach to investigate complex traits, originally predominantly used in human disease research (1). With the maturation of sequencing technologies, complex traits in livestock and poultry have increasingly become the focus of GWAS studies. Currently, the application of GWAS techniques plays a significant role in studying quantitative trait loci (QTLs), single nucleotide polymorphisms (SNPs), and candidate functional genes associated with complex economic traits in livestock and poultry. It has been extensively employed in molecular genetic marker selection in poultry. For instance, Zhu et al. conducted a GWAS analysis on body size and carcass traits in Beijing ducks, discovering specific mutation sites in the NR2F2 gene related to high skin fat content in Beijing ducks. They also identified a genome-wide significant locus associated with carcass weight and eviscerated weight among five body composition traits, with ATP11A being the candidate gene (2, 3). Additionally, a researcher performed a single-haplotype GWAS study using a 60 K SNP chip in two specialized meat chicken strains with divergent abdominal fat content, resulting in the localization of seven candidate genes potentially regulating abdominal fat content, including SHH, LMBR1, IGF1R, and SLC16A (4).

The body size traits in avian species, such as semi-aquatic length and body slant length, directly influence their physiological adaptability, with larger body size typically indicating stronger skeletal and muscular systems. Given that body size traits in geese are complex quantitative traits influenced by multiple factors, the heritability of traits such as semi-aquatic length, neck length, and body slant length is relatively high. Genetic analysis can be employed to identify major-effect gene regions associated with these traits. Therefore, the objective of this study is to conduct a genome-wide association study (GWAS) to identify molecular markers related to body size traits in geese and localize functional gene regions, thus providing theoretical support for elucidating the genetic mechanisms underlying body size traits and facilitating marker-assisted selection.

## 2 Materials and methods

### 2.1 Ethics statement

The experimental protocol was in accordance with the guidelines on the care and use of experimental animals issued by the State Council of the People’s Republic of China (Approval number:2006–398). In addition, the animal experiments were approved by the Animal Management and Ethics Committee of the Jiangsu Agri-Animal Husbandry Vocational College (Permit No. jsahvc-2022-32), and all experimental procedures strictly followed the related laboratory regulations and the relevant guidelines.

### 2.2 Populations and phenotype

In this study, we conducted a random selection of hatching eggs from five Chinese local goose populations in the National Waterfowl Gene Bank (Jiangsu). These eggs were incubated under uniform conditions until reaching 10 weeks of age. A total of 251 samples were measured for body size data, including 50 individuals of Taihu (TH) geese (25 males and 25 females), 47 individuals of Guangfeng (GF) geese (23 males and 24 females), 49 individuals of Sichuan (SC) geese (24 males and 25 females), 47 individuals of Xupu (XP) geese (23 males and 24 females), and 58 individuals of Youjiang (YJ) geese (30 males and 28 females). Subsequently, approximately 5 mL of blood was collected from each bird’s sublingual vein using blood collection tubes containing EDTA-K2 anticoagulant. The geese were released after blood sampling. At 10 weeks of age, 9 body size measurements, including body Semi-submerged length (SSL):the distance from the tip of the mouth to the midpoint of the hip connection; Neck length (NL): the distance from the anterior edge of the first cervical vertebra to the base of the neck; Body oblique length (BOL): the distance between the shoulder joint and the ischial tuberosity is measured on the body surface; Keel length (KL): the distance from the front end of the keel process to the end of the keel on the body surface; Shank circumference (SC): the circumference of the middle tibia; Chest width (CW): the body surface distance between the shoulder joints with calipers; Chest depth (CD): the distance from the first thoracic vertebra to the anterior edge of the keel on the body surface with calipers; Pelvis width (PW): the distance between the two hip tuberosities with calipers; Shank length (SL): the straight-line distance from the superior tibial joint to the third and fourth toes were. All the measure methods were following the guidelines outlined in the third national resource census measurement manual.

### 2.3 Genotype and quality control

Samples of whole blood (5 mL) collected via the jugular vein were placed in vacuum containers containing K2EDTA as an anticoagulant. Genomic DNA from the samples was extracted using the phenol-chloroform method (5). The sequencing method of PE150 was selected using the DNBSEQ-T7 sequencer for machine sequencing. The FastQC software was utilized to conduct a comprehensive quality assessment of the raw data:

1. Fragments containing adapter sequences were filtered out.
2. Paired fragments were removed if the proportion of N bases in single-end reads exceeded 10% of the fragment length.
3. Paired fragments were discarded if the number of low-quality bases (≤5) in single-end sequencing reads exceeded 50% of the fragment length.

The high-quality sequencing data were aligned to the goose chromosome-level reference genome version (6), using the Burrows-Wheeler Aligner (BWA) software (7) with the parameters: mem-t 4-K 32-M. Duplicates were eliminated using SAMtools with the rmdup parameter (8). Finally, the alignment rate of the samples was analyzed and calculated.

The depth of coverage for each sample was calculated by averaging the number of aligned reads across the entire genome using SAMtools. SAMtools was employed to detect SNPs in the population samples using the following filtering and selection methods, resulting in the identification of high-quality SNPs: SNPs were supported by a coverage depth greater than 2; The proportion of missing genotypes was less than 10%; The minimum allele frequency (MAF) was set at 5%.

### 2.4 Genome-wide association studies

In this study, we employed the multi-locus mixed linear model, known as FarmCPU (9), to conduct a genome-wide association analysis based on multiple markers for nine phenotypic traits in 10-week-old geese. The specific fixed-effects model used in the association analysis is described as follows:

Fixed effects model within FarmCPU:


$$y=Pb_{p}+M_{t}b_{t}+S_{j}d_{j}+e$$

y: the phenotypic values; P: the fixed effects (gender, the first three principal components, and breed); M_t::_ the fixed effects of t pseudo-QTNs; b_p:_ the matrix accounted for fixed effect mapping; b_t:_ M_t_ mapping matrix; S_j:_ the j-th mark to be tested; d_j:_ S_j_ mapping matrix; e: a residual vector with a normal distribution e~N (0, Iσ_e_^2^), I is the identity matrix, σ_e_^2^ is the unknown residual.

Random-effects model in the FarmCPU model (used to select pseudo-QTN):


y=u+e

y: the phenotypic values; u: Var(u) = σ_g_^2^K random effects, K is the kinship matrix; e:a residual vector with a normal distribution e~N (0, Iσ_e_^2^), Iis the identity matrix, σ_e_^2^ is the unknown residual.

The Bonferroni correction threshold for multiple tests, which was employed for detecting the genome-wise significant SNPs, was defined as *p* = *α*/N (*α* = 0.05, N is the number of SNPs). The suggestive significant threshold was set at *p* = 5.00E-07 in this study (10).

### 2.5 Candidate gene functional annotation

To establish a comprehensive gene annotation database for geese, we employed the annovar software following the methodology outlined (11). Subsequently, the SNPs that exhibited significant associations with various phenotypic traits in 10-week-old geese were subjected to gene annotation. The gene annotation process involved assigning functional annotations to these SNPs within a specified genomic region spanning 20 kb upstream and downstream of the SNP loci deemed significant. Furthermore, to elucidate the functional roles of the identified candidate genes and their involvement in signaling pathways, we conducted Gene Ontology (GO) and Kyoto Encyclopedia of Genes and Genomes (KEGG) pathway enrichment analyses using the KOBAS 3.0 online tool (12).

## 3 Results

### 3.1 Summary information of phenotypic data and genotypic data

A total of 251 individual samples were included in the analysis of phenotypic data, fulfilling the data requirements for population genetics analysis. Descriptive statistics of nine body size traits in 10-week-old geese from distinct geographical regions are presented in Table 1. The findings reveal that the Right River goose population exhibited the smallest body size among the five populations, with a minimum semi-submerged length trait value of 51.07 cm. In contrast, the other four populations displayed relatively comparable body sizes, with an average around 54 cm for this particular trait. Additionally, concerning the NL, BOL, KL, SC, CW, and CD traits, the Right River goose population consistently demonstrated lower body size measurements compared to the other four populations. Conversely, for the PW and SL traits, the five populations exhibited similar phenotypic values, with mean measurements of 7.37 and 10.78 cm, respectively, indicating minimal differences among the populations.

**Table 1:** The descriptive statistical of 9 body size traits for all breeds.

Breed	SSL	NL	BOL	KL	SC	CW	CD	PW	SL	NO
TH	54.66 ± 2.81	24.42 ± 2.44	26.36 ± 1.81	14.67 ± 1.26	4.73 ± 0.25	8.96 ± 0.98	7.76 ± 0.93	7.34 ± 0.64	10.82 ± 0.80	50
GF	54.07 ± 3.47	24.53 ± 2.26	26.1 ± 3.29	14.19 ± 1.25	4.87 ± 0.34	10.1 ± 0.88	8.24 ± 0.72	7.27 ± 0.52	11.14 ± 0.65	47
SC	54.83 ± 3.67	23.86 ± 2.92	27.97 ± 3.20	14.62 ± 1.64	4.92 ± 0.34	9.62 ± 0.81	8.00 ± 0.84	7.83 ± 0.53	10.66 ± 0.31	49
XP	54.59 ± 3.23	24.34 ± 2.49	28.26 ± 2.13	14.85 ± 1.18	4.86 ± 0.22	9.8 ± 1.37	8.38 ± 1.17	7.23 ± 0.75	10.92 ± 0.81	47
YJ	51.07 ± 3.41	21.61 ± 2.57	25.24 ± 2.62	12.74 ± 1.27	4.48 ± 0.24	8.93 ± 0.88	7.79 ± 0.88	7.19 ± 0.41	10.42 ± 0.63	58
ALL	53.74 ± 3.62	23.67 ± 2.78	26.72 ± 2.89	14.16 ± 1.54	4.76 ± 0.33	9.45 ± 1.09	8.02 ± 0.94	7.37 ± 0.62	10.78 ± 0.70	251

SSL, semi-submerged length; NL, neck length; BOL, body oblique length; KL, keel length; SC, shank circumference; CW, chest width; CD, chest depth; PW, pelvis width; SL, shank length.

**Figure 1:**
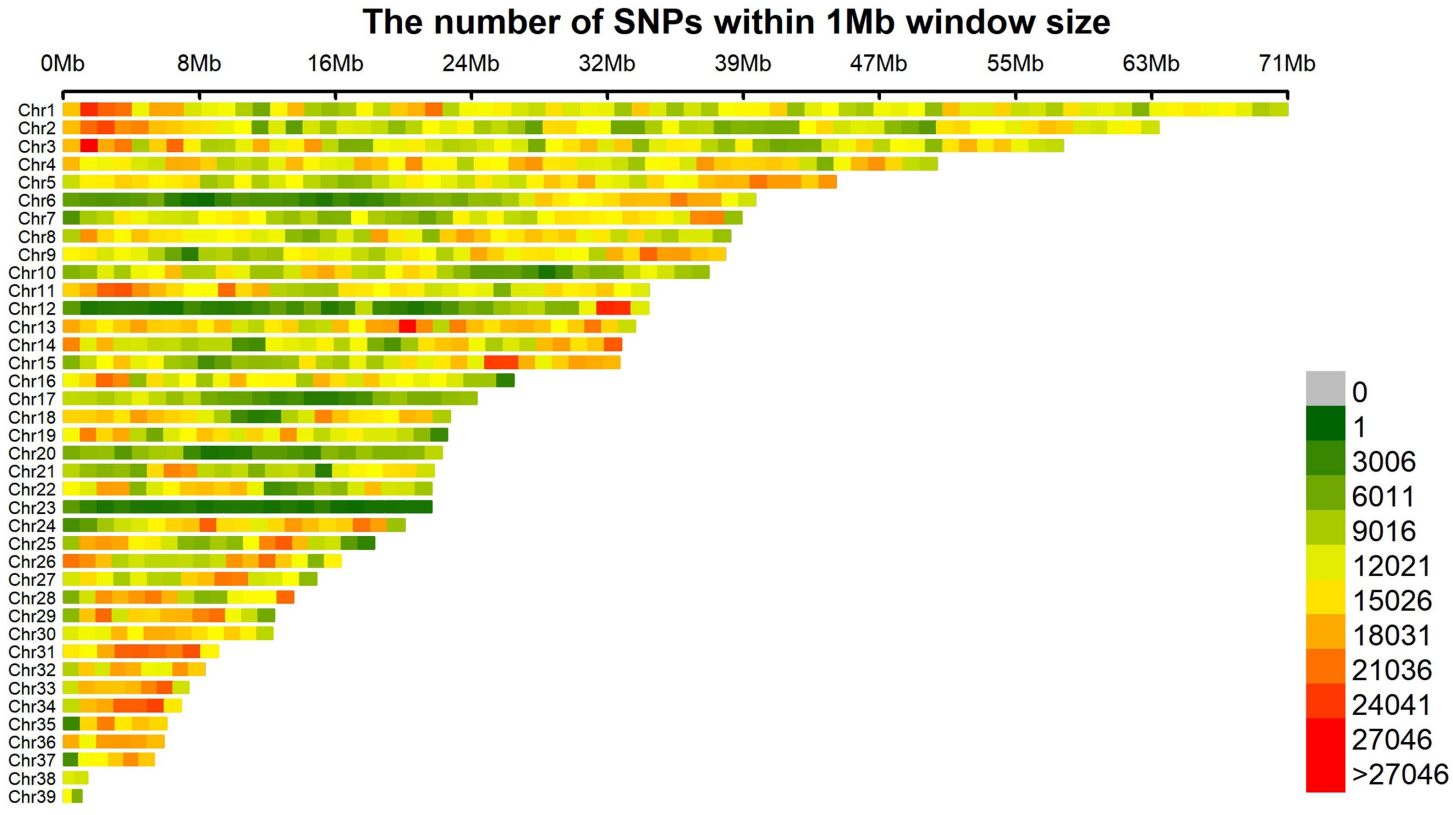
The genome-wide distribution density map of SNPs.

The sequencing results are presented in Table 2, demonstrating an average raw data yield of 23.91 Gb per sample during the sequencing process. After applying quality control measures, an average of 23.46 Gb of clean data was obtained per sample. The evaluation of sequencing quality indicated a remarkable average Q20 value of 98.08% across all samples, indicating high-quality sequencing data suitable for re-sequencing purposes. The mean GC content of 42.87 fell within the normal range, signifying successful construction of the sequencing libraries in this study. Notably, the sequence alignment statistics revealed an impressive average alignment rate of 98.60% across the samples, accompanied by an average sequencing depth of 19.78X, which denotes a robust capacity for detecting genetic variations. Following stringent detection and quality control procedures, a total of 12,187,196 SNP loci were retained for subsequent genome-wide association analysis of body size traits in 251 samples at the age of 10 weeks. The distribution pattern of SNP density within 1 Mb windows on autosomes after quality control is visually depicted in Figure 1.

**Table 2:** The statistical table of sample sequencing data quality control and sequence alignment.

Breed	Raw base (bp)	Clean base (bp)	Effective ratio (%)	GC rate (%)	Q20 (%)	Mapping rate	Depth
TH	24,016,411,224 ± 9,189,778,298	23,563,532,076 ± 8,970,587,798	98.19 ± 1.72	42.80 ± 0.39	97.95 ± 0.65	98.45 ± 0.97	21.25 ± 7.82
GF	24,817,858,934.04 ± 9,096,634,473.59	24,328,986,906.38 ± 9,007,748,701.52	97.98 ± 2.52	42.84 ± 0.26	98.43 ± 0.41	98.66 ± 0.13	20.74 ± 7.95
SC	24,127,459,858.82 ± 9,265,408,060.40	23,646,575,752.94 ± 9,047,621,436.39	98.05 ± 1.98	42.75 ± 0.27	98.37 ± 0.41	98.59 ± 0.14	20.65 ± 7.94
XP	24,767,606,656.25 ± 9,368,470,498.39	24,253,905,925.31 ± 9,250,685,134.96	97.86 ± 2.16	42.85 ± 0.42	97.88 ± 1.19	98.63 ± 0.15	21.18 ± 8.02
YJ	22,252,817,186.44 ± 8,583,711,329.31	21,873,614,933.89 ± 8,366,239,017.01	98.46 ± 2.01	43.08 ± 0.34	97.83 ± 1.63	98.61 ± 0.12	19.04 ± 7.11
ALL	23,919,693,009.41 ± 9,083,820,257.11	23,460,176,930.59 ± 8,906,156,249.17	98.13 ± 2.08	42.87 ± 0.36	98.08 ± 1.04	98.60 ± 0.41	19.78 ± 7.44

### 3.2 GWAS on 9 body size traits for the aggregated dataset

Our study employed the FarmCPU multi-locus mixed linear model to perform a comprehensive genome-wide association analysis of nine body size traits in 10-week-old geese. Given that genotyping was accomplished through re-sequencing, the resulting dataset consisted of a substantial number of SNP loci, totaling 12,187,196. Applying the Bonferroni correction method for determining significance thresholds would be overly conservative under these circumstances. Consequently, our study adopted two threshold lines: a significant threshold line (*p* = 0.05 / N, *p* = 3.98E-09) and an empirical threshold line (*p* = 5E-07), to identify SNP loci significantly associated with the investigated body size traits.

**Figure 2:**
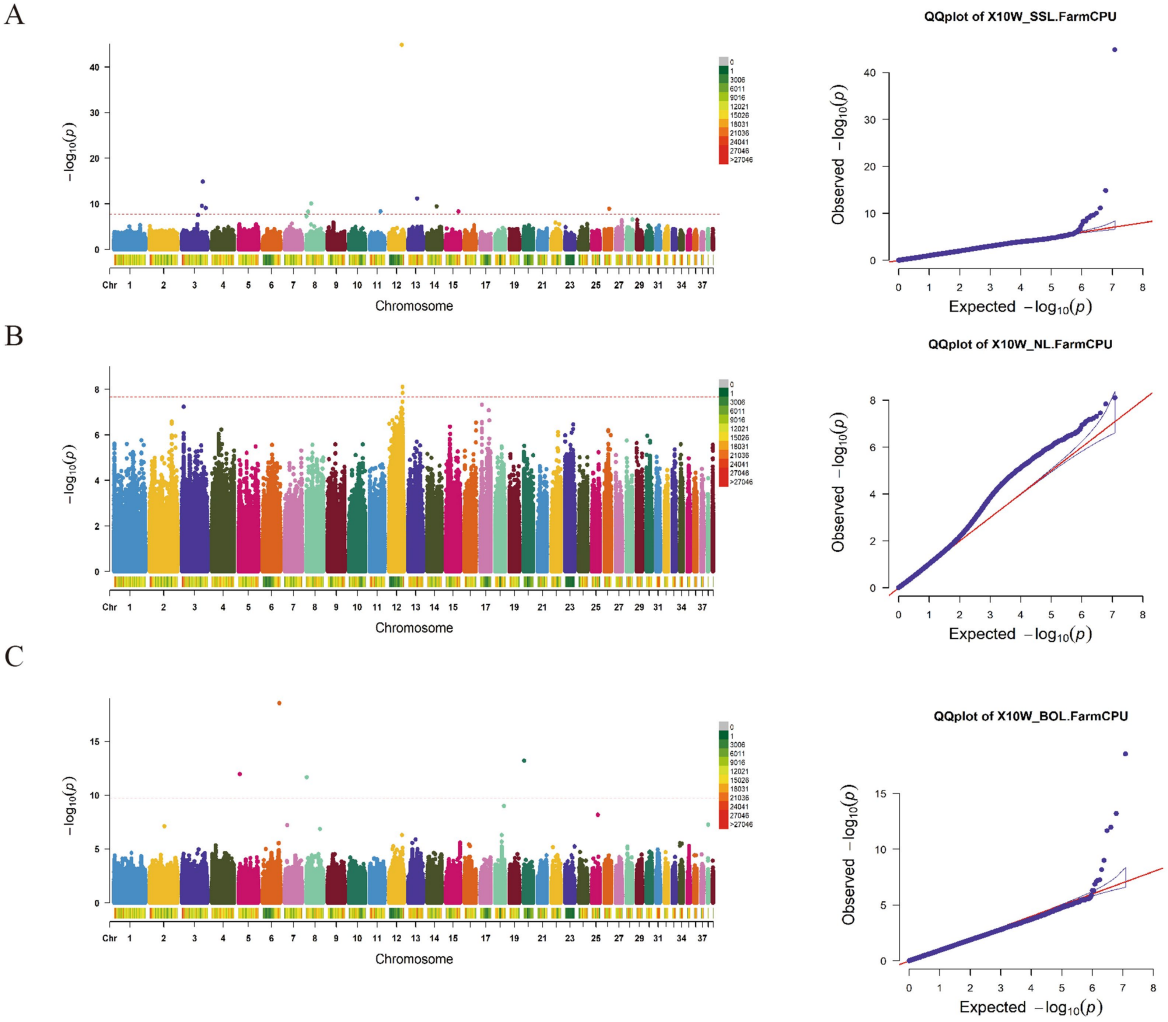
The manhattan plots and Q-Q diagram of SSL **(A)**, NL **(B)** and BOL **(C)** of 10-week-old geese.

The GWAS results for the SSL, NL, and BOL traits (Figure 2; Supplementary Table S1) reveal the identification of 12 significant loci associated with the SSL trait. Among these, one SNP locus (Chr3:45120340) is situated downstream of the PUR8 and ADSL genes, while another SNP locus (Chr3:36533197) is located upstream of the OSTCN and MGP genes. Moreover, on Chr27, one SNP locus (Chr27:13604716) was found within an exon region of the THADA gene. Concerning the neck length trait, a total of 41 loci are significantly associated. Within this set, three SNP loci reside upstream of the PIAS2 gene on Chr12, whereas five SNP loci are positioned downstream. Additionally, one SNP locus (Chr23:18060660) on Chr23 and another SNP locus (Chr15:9095561) on chromosome 15 are located within the exon regions of the ZNF462, PRDM8, and GH14523 genes, respectively. Notably, one SNP (Chr2:50292822) on Chr2 corresponds to a variable splicing site of the NONE gene. Regarding the body oblique length trait, a total of 7 SNP loci have been identified. Among them, one SNP locus at position 33,451,557 on Chr2 is situated in the upstream region of the ACOT1 gene.

**Figure 3:**
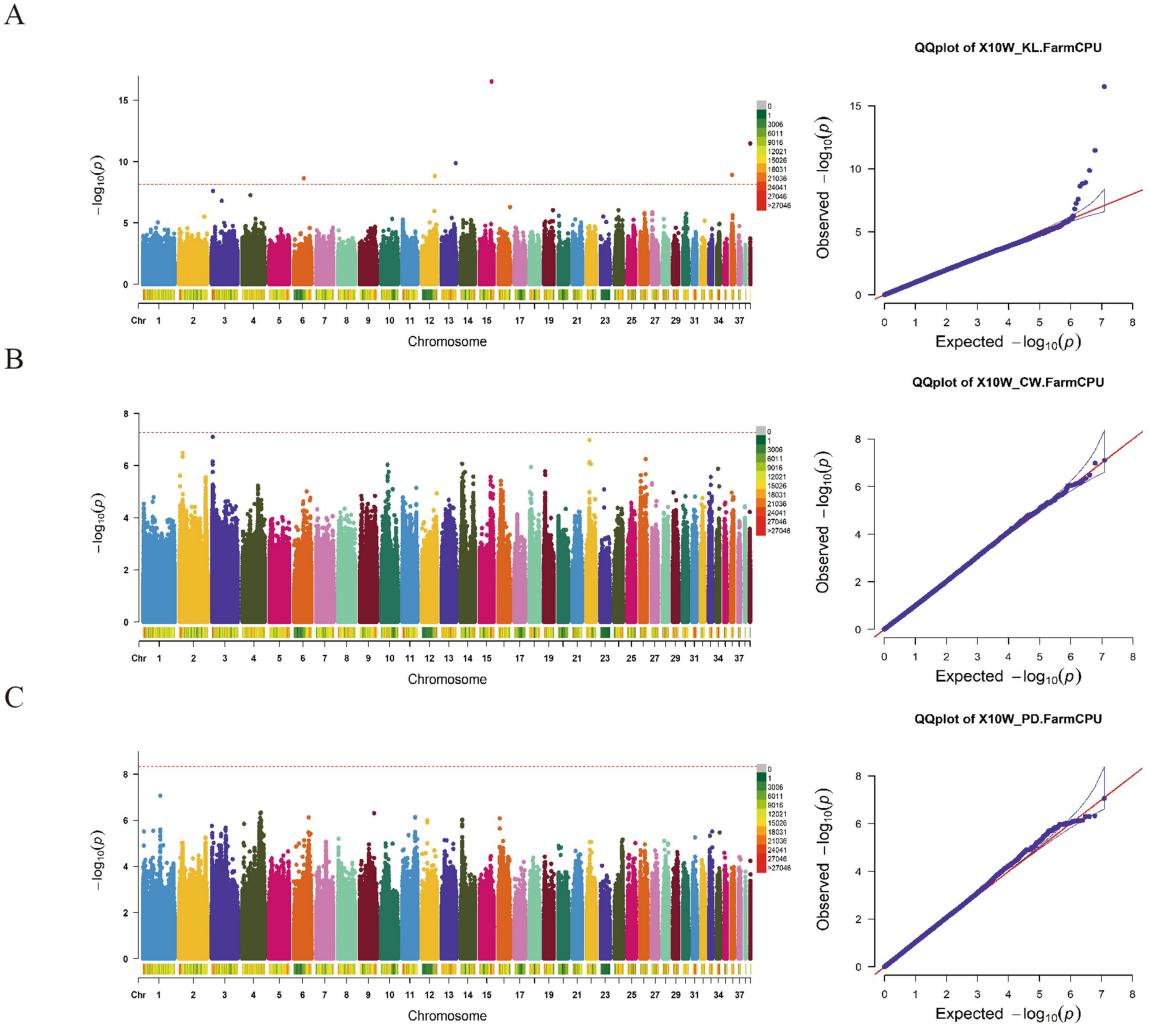
The manhattan plots and Q-Q diagram of KL **(A)**, CW **(B)** and CD **(C)** of 10-week-old geese.

The GWAS results for the KL, CW, and CD traits (Figure 3; Supplementary Table S1) unveiled a total of eight significant SNP loci. Among these, 4 SNP loci exhibited associations with the keel length trait. Specifically, 2 SNP loci (Chr15:26888407 and Chr6:22659825) were situated within intronic regions of the CTNNA2 and TUSC3 genes, respectively. Regarding the CW trait, 2 SNP loci on Chr2 were identified, both residing within intronic regions of the TF3B and BRF1 genes. Additionally, 2 SNP loci (Chr1:39289602 and Chr4:40609098) demonstrated significant associations with the CD trait, positioned within intronic regions of the AIM1, OR56B2P, and SULT1C4 genes, respectively.

**Figure 4:**
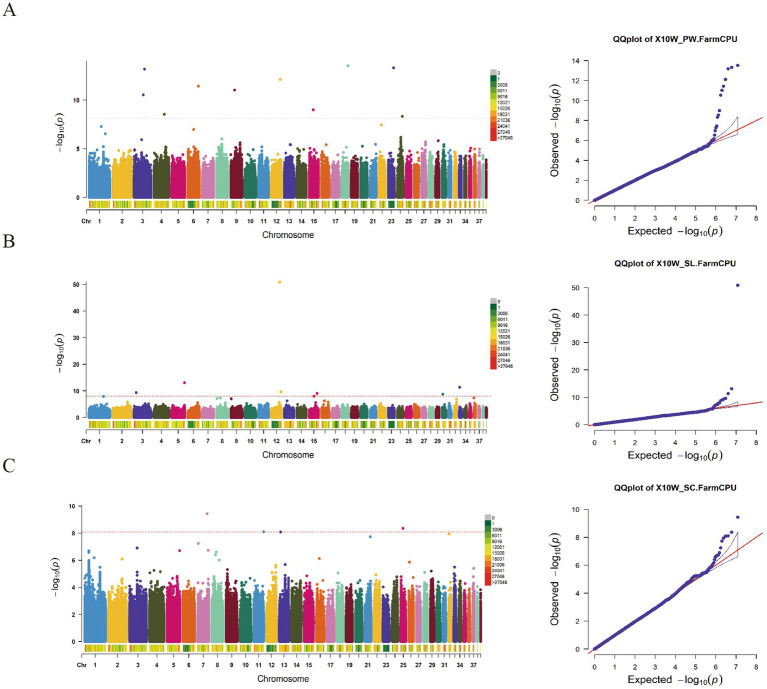
The manhattan plots and Q-Q diagram of PW **(A)**, SL **(B)** and SC **(C)** of 10-week-old geese.

The GWAS results for the PW, SL, and SC traits (Figure 4; Supplementary Table S1) identified a total of 24 significant SNP loci. Among these, 6 loci exhibited associations with the PW trait. Specifically, 4 SNP loci (Chr23:18669625, Chr22:8080154, Chr1:41472846, and Chr1:54957903) were located within intronic regions of the LMNB1, GCP, DPOLZ, REV3L, and AR1D1B genes. Regarding the SL trait, 2 SNP loci (Chr33:831318 and Chr36:52899) were found within intronic regions of the MACF1 and SH3R3 genes, respectively. Concerning the SC trait, 14 SNP loci were associated, with one SNP locus (Chr11:33550276) located downstream of the NECAP1 gene, and 7 SNP loci residing within intronic regions of various genes.

**Figure 5:**
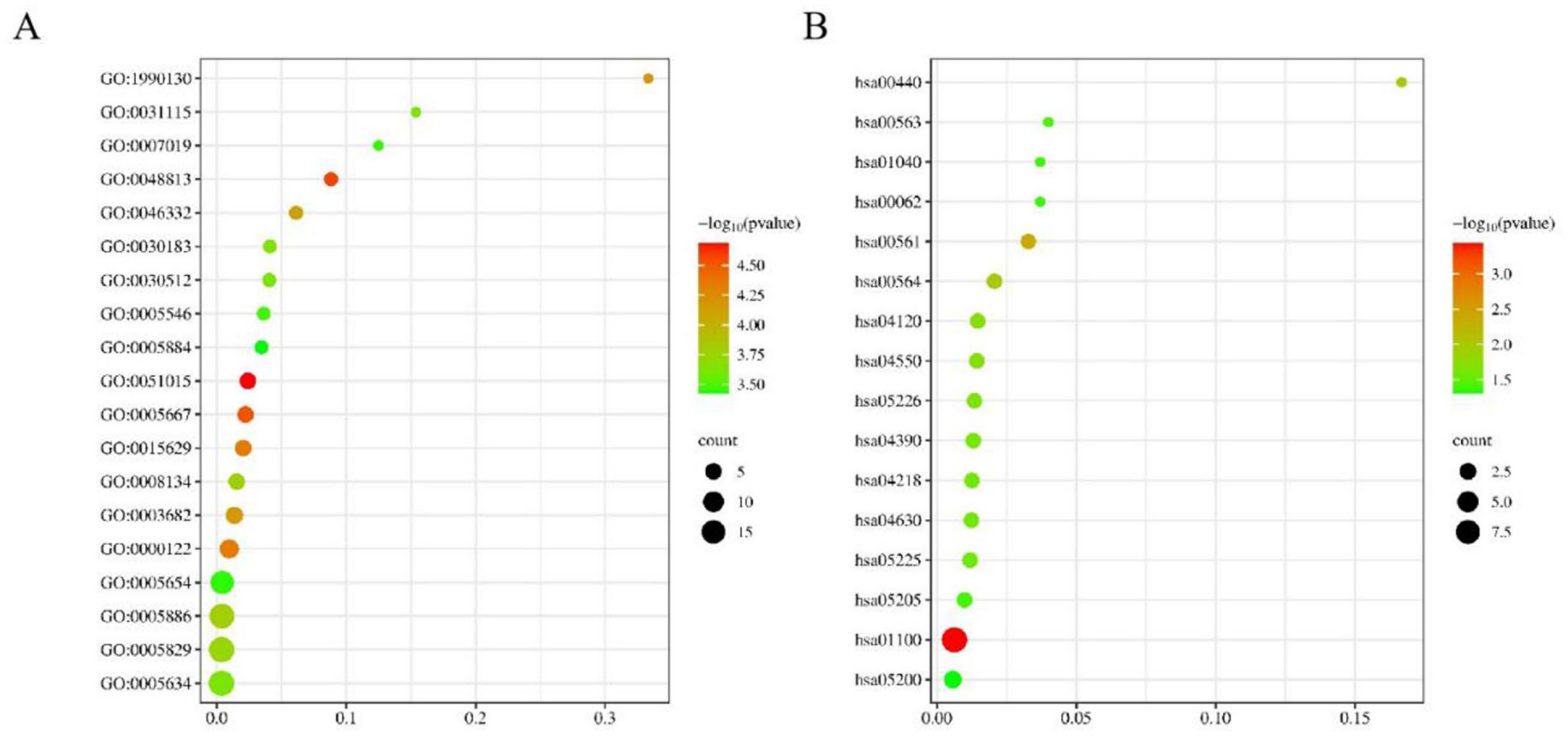
The bubble plot for candidate gene GO **(A)** and KEGG **(B)** enrichment analysis.

In our study, a comprehensive enrichment analysis was conducted on a set of 86 candidate genes identified from the GWAS results associated with 9 body size traits in 10-week-old geese. The results of the enrichment analysis were presented in Tables 3, 4. As shown in Table 3 and Figure 5, several GO terms related to cellular and skeletal development, such as ‘the actin filament binding (GO:0051015) pathway’ and ‘the negative regulation of transforming growth factor beta receptor signaling pathway (GO:0030512)’ were significantly associated with body size traits. These terms are critical in understanding the molecular pathways that regulate growth and development, which in turn influence body size. In the KEGG enrichment analysis, the Metabolic pathways (hsa01100) pathway emerged as the most significantly enriched pathway, involving a total of 9 candidate genes: DNMT3B, TUSC3, CMPK2, EPT1, AGPAT3, ADSL, GK, ATP5A1, and ACOT1.

**Table 3:** The GO enrichment analysis results of candidate genes related to body size traits.

Term	ID	Gene number	*p*-value
Actin filament binding	GO:0051015	5	2.07E-05
Dendrite morphogenesis	GO:0048813	3	2.66E-05
Transcription regulator complex	GO:0005667	5	3.06E-05
Actin cytoskeleton	GO:0015629	5	4.45E-05
Negative regulation of transcription by RNA polymerase II	GO:0000122	8	4.56E-05
GATOR1 complex	GO:1990130	2	6.59E-05
Chromatin binding	GO:0003682	6	7.02E-05
SMAD binding	GO:0046332	3	7.45E-05
Plasma membrane	GO:0005886	18	1.63E-04
Transcription factor binding	GO:0008134	5	1.64E-04
Cytosol	GO:0005829	19	1.80E-04
B cell differentiation	GO:0030183	3	2.31E-04
Nucleus	GO:0005634	19	2.40E-04
Negative regulation of transforming growth factor beta receptor signaling pathway	GO:0030512	3	2.40E-04
Negative regulation of microtubule polymerization	GO:0031115	2	2.46E-04
Phosphatidylinositol-4,5-bisphosphate binding	GO:0005546	3	3.32E-04
Microtubule depolymerization	GO:0007019	2	3.57E-04
Nucleoplasm	GO:0005654	15	3.58E-04
Actin filament	GO:0005884	3	3.80E-04

**Table 4:** The KEGG enrichment analysis results of candidate genes related to body size traits.

Term	ID	Gene number	*p*-value
Metabolic pathways	hsa01100	9	3.65E-04
Glycerolipid metabolism	hsa00561	2	4.35E-03
Glycerophospholipid metabolism	hsa00564	2	1.04E-02
Phosphonate and phosphinate metabolism	hsa00440	1	1.08E-02
Ubiquitin mediated proteolysis	hsa04120	2	1.98E-02
Signaling pathways regulating pluripotency of stem cells	hsa04550	2	2.06E-02
Gastric cancer	hsa05226	2	2.31E-02
Hippo signaling pathway	hsa04390	2	2.46E-02
Cellular senescence	hsa04218	2	2.64E-02
Jak–STAT signaling pathway	hsa04630	2	2.70E-02
Hepatocellular carcinoma	hsa05225	2	2.88E-02
Glycosylphosphatidylinositol (GPI)-anchor biosynthesis	hsa00563	1	3.96E-02
Proteoglycans in cancer	hsa05205	2	4.05E-02
Biosynthesis of unsaturated fatty acids	hsa01040	1	4.26E-02
Fatty acid elongation	hsa00062	1	4.26E-02
Pathways in cancer	hsa05200	3	5.01E-02

## 4 Discussion

The body size traits of geese at 10 weeks of age play a pivotal role in their productivity and economic viability. We reviewed recent studies on chicken morphometric traits (13) and highlighted traits such as chest width, chest depth and shank length, which are commonly used to predict growth rates and meat production in chickens. These traits serve as direct indicators of the overall growth and development of geese. Larger SSL, NL, and BOL are often associated with robust skeletal and muscular systems, reflecting enhanced growth potential and physical health in geese. It is noteworthy that body size traits also exhibit close correlations with slaughter performance and meat quality characteristics in geese. Increased CD, for instance, is closely linked to the development of chest muscles, which is of utmost importance for improving meat quality and yield in geese. Additionally, SSF and BOL influence the cutting parts and texture of goose meat, thereby impacting its nutritional value and sensory experience. For goose breeders, the identification of molecular markers and candidate genes associated with body size traits through genome-wide association analysis holds significant value in unraveling the underlying mechanisms governing these traits.

In our study, we selected 5 representative local goose breeds from China and measured nine phenotypic traits related to body size in a sample comprising 251 individuals. Genotyping data were collected, and the average sequencing depth per sample reached 19.78X. Subsequently, employing the FarmCPU multi-locus genome-wide association analysis method based on 12,187,196 SNP loci, we identified 92 SNP loci significantly associated with the 9 body size traits. Following gene annotation, these SNP loci were mapped to a total of 86 candidate genes. To gain further insights into the functional roles of these candidate genes, we conducted GO and KEGG enrichment analyses, resulting in the identification of 36 significantly enriched pathways. The molecular markers and candidate genes discovered through this study provide valuable reference data for guiding goose breeding programs and deepening our understanding of the genetic mechanisms underlying goose growth and development. Subsequent sections of this paper will delve into a detailed discussion of select candidate genes and key signaling pathways identified in our study.

In this study, we identified a significant single nucleotide polymorphism (SNP) (Chr27:13604716) located within the exon region of the Thyroid Adenoma Associated gene (*THADA*) on Chr27, which showed a significant association with the semi-submerged length trait in geese. This SNP may potentially impact body size variation by exerting functional effects on the *THADA* gene. The precise role of *THADA* in growth and development is still under investigation; however, previous studies have suggested associations between *THADA* gene variants and growth-related traits, including body weight, in humans and animals (14).

Furthermore, we identified a significant SNP (Chr23:18060660) located within the exon region of the *ZNF462* gene on Chr23, which demonstrated a significant association with the neck length trait. This SNP might influence body size variation by modulating the function of the *ZNF462* gene. The protein encoded by *ZNF462* belongs to the C2H2-type zinc finger family of transcription factors and has been implicated in transcriptional regulation. While the precise functions of *ZNF462* are not fully elucidated, studies have indicated its involvement in early embryonic development (15). Notably, *ZNF462* mutations have been associated with growth hormone deficiency in Wiedemann-Steiner syndrome patients (16), and investigations in Chinese Simmental beef cattle have linked the *ZNF462* gene to body weight traits (17).

Moreover, we identified a significant SNP (Chr15:9095561) located within the exon region of the *PRDM8* gene on Chr15, which exhibited a significant association with the neck length trait. This SNP could potentially impact body size variation by influencing the function of the *PRDM8* gene. *PRDM8* encodes a protein belonging to the PR domain-containing family of histone methyltransferases, primarily acting as negative transcriptional regulators (18, 19). Studies employing *PRDM8* knockout mice have demonstrated growth retardation upon its deletion (20). These findings collectively suggest that the *PRDM8* gene may play a role in influencing the growth and development of geese.

We conducted functional enrichment analysis by compiling the candidate genes associated with the nine body size traits into a consolidated dataset. The identified GO terms (Table 3) provide valuable insights into the molecular mechanisms underlying body size in geese. For instance, the “negative regulation of transforming growth factor beta receptor signaling pathway (GO:0030512)” and “Signaling pathways regulating pluripotency of stem cells (hsa04550)” are likely to play crucial roles in the growth and development of body size in geese. Transforming growth factor beta (*TGF-β*) and its related factors are known regulators of cell proliferation, differentiation, apoptosis, and migration, thereby influencing developmental processes and tissue homeostasis (21). Previous studies have reported associations between *TGF-β* genes and growth and development in chickens, impacting body weight and chest muscle weight (22).

The *SMAD2* gene, which is shared by both pathways, plays a pivotal role in the formation of protein complexes involving TGF-β and bone morphogenetic proteins (BMPs). Smads proteins function as transcription factors and mediate signal transduction within the TGF-β and BMP signaling pathways. They exert regulatory effects on osteoblast and osteoclast functions, thus critically influencing bone remodeling processes (23). While specific investigations on the involvement of the *SMAD2* gene in goose skeletal development are currently lacking, we speculate that it holds a key and indispensable role in the determination of body size in geese. Therefore, further research is warranted to elucidate its precise function.

While the current study, based on 251 samples, provides robust evidence of the large effects of certain morphometric traits on growth performance, it is important to acknowledge that smaller effects, which may exist, were not detectable within the scope of this sample size. Future research with larger sample sizes will be crucial to explore the full spectrum of variation in these traits.

## 5 Conclusion

Our study employed re-sequencing data to conduct a genome-wide association study (GWAS) using the FarmCPU model on 9 body size traits in 5 Chinese indigenous goose breeds. The analysis revealed 88 significant SNPs associated with body size traits, including 16 SNPs surpassing the genome-wide significance threshold (*p* = 3.98E-09) and 72 SNPs exceeding the suggestive significance threshold (*p* = 5E-07). Subsequent gene annotation identified these SNPs to be located within exonic regions of 86 candidate genes. Importantly, functional enrichment analysis utilizing GO and KEGG pathways revealed the “negative regulation of transforming growth factor beta receptor signaling pathway” (GO:0030512) emerged as particularly relevant to goose skeletal development and the phenotypic expression of body size in geese. These findings contribute valuable insights into the genetic architecture of body size traits in 10-week-old geese and provide a foundation for future molecular breeding efforts and the elucidation of underlying mechanisms governing body size trait formation in geese.

## Data Availability

The genome datasets presented in this study can be found in the CNCB-NGDC repository, accession numbers PRJCA019438.

## References

[1] Uffelmann E Huang QQ Munung NS de Vries J Okada Y Martin AR. Genome-wide association studies. Nat Rev Methods Primers. (2021) 1:59. doi: 10.1038/s43586-021-00056-9

[2] Zhu F Cui QQ Hou ZC. SNP discovery and genotyping using genotyping-by-sequencing in Pekin ducks. Sci Rep. (2016) 6:36223. doi: 10.1038/srep36223 27845353 PMC5109183

[3] Zhu F Yin ZT Wang Z Smith J Zhang F Martin F. Three chromosome-level duck genome assemblies provide insights into genomic variation during domestication. Nat Commun. (2021) 12:5932. doi: 10.1038/s41467-021-26272-1 34635656 PMC8505442

[4] Zhang H Shen LY Xu ZC Kramer LM Yu JQ Zhang XY. Haplotype-based genome-wide association studies for carcass and growth traits in chicken. Poult Sci. (2020) 99:2349–61. doi: 10.1016/j.psj.2020.01.009 32359570 PMC7597553

[5] Galvis AE Fisher HE Camerini D. NP-40 fractionation and nucleic acid extraction in mammalian cells. Bio Protoc. (2017) 7:e2584. doi: 10.21769/BioProtoc.2584 PMC8438428 34595266

[6] Yan L Guangliang G Yu L Silu H Yi L Guosong W. Pacific biosciences assembly with hi-C mapping generates an improved, chromosome-level goose genome. GigaScience. (2020) 9:1–8. doi: 10.1093/gigascience/giaa114 PMC7585555 33099628

[7] Li H. Aligning sequence reads, clone sequences and assembly contigs with BWA-MEM. arXiv: Genomics. (2013). doi: 10.48550/arXiv.1303.3997

[8] Li H Handsaker B Wysoker A Fennell T Ruan J Homer N. 1000 genome project data processing subgroup. The sequence alignment/map format and SAMtools. Bioinformatics. (2009) 25:2078–9. doi: 10.1093/bioinformatics/btp352 19505943 PMC2723002

[9] Liu X Huang M Fan B Buckler ES Zhang Z. Iterative usage of fixed and random effect models for powerful and efficient genome-wide association studies. PLoS Genet. (2016) 12:e1005767. doi: 10.1371/journal.pgen.1005767 26828793 PMC4734661

[10] Panagiotou OA Ioannidis JPA Hirschhorn JN Abecasis GR Frayling TM McCarthy MI. What should the genome-wide significance threshold be? Empirical replication of borderline genetic associations. Int J Epidemiol. (2012) 41:273–86. doi: 10.1093/ije/dyr178 22253303

[11] Wang K Li M Hakonarson H. ANNOVAR: functional annotation of genetic variants from high-throughput sequencing data. Nucleic Acids Res. (2010) 38:e164. doi: 10.1093/nar/gkq603 20601685 PMC2938201

[12] Bu D Luo H Huo P Wang Z Zhang S He Z. KOBAS-i: intelligent prioritization and exploratory visualization of biological functions for gene enrichment analysis. Nucleic Acids Res. (2021) 49:W317–25. doi: 10.1093/nar/gkab447 34086934 PMC8265193

[13] Tadele A Berhane G Esatu W Wassie T. Effect of genotype on hatchability, growth, morphometric and carcass traits of chicken. J Agric Food Res. (2023) 11:100531. doi: 10.1016/j.jafr.2023.100531

[14] Gupta V Vinay DG Sovio U Rafiq S Kranthi Kumar MV Janipalli CS. Association study of 25 type 2 diabetes related loci with measures of obesity in Indian sib pairs. PLoS One. (2013) 8:e53944. doi: 10.1371/journal.pone.0053944 23349771 PMC3547960

[15] Laurent A Masse J Omilli F Deschamps S Richard-Parpaillon L Chartrain I. ZFPIP/Zfp462 is maternally required for proper early *Xenopus laevis* development. Dev Biol. (2009) 327:169–76. doi: 10.1016/j.ydbio.2008.12.005 19111535

[16] Zhou Y Liu J Wu S Li W Zheng Y. Case report: a heterozygous mutation in ZNF462 leads to growth hormone deficiency. Front Genet. (2022) 13:1015021. doi: 10.3389/fgene.2022.1015021 36568367 PMC9770794

[17] Du L Duan X An B Chang T Liang M Xu L. Genome-wide association study based on random regression model reveals candidate genes associated with longitudinal data in Chinese Simmental beef cattle. Animals. (2021) 11:2524. doi: 10.3390/ani11092524 34573489 PMC8470172

[18] Fog CK Galli GG Lund AH. PRDM proteins: important players in differentiation and disease. BioEssays. (2012) 34:50–60. doi: 10.1002/bies.201100107 22028065

[19] Hohenauer T Moore AW. The Prdm family:expanding roles in stem cells and development. Development. (2012) 139:2267–82. doi: 10.1242/dev.070110 22669819

[20] Inoue M Iwai R Yamanishi E Yamagata K Komabayashi-Suzuki M Honda A. Deletion of Prdm8 impairs development of upper-layer neocortical neurons. Genes Cells. (2015) 20:758–70. doi: 10.1111/gtc.12274 26283595

[21] Chen YG Meng AM. Negative regulation of TGF-beta signaling in development. Cell Res. (2004) 14:441–9. doi: 10.1038/sj.cr.7290246 15625010

[22] Li H Deeb N Zhou H Mitchell AD Ashwell CM Lamont SJ. Chicken quantitative trait loci for growth and body composition associated with transforming growth factor-beta genes. Poult Sci. (2003) 82:347–56. doi: 10.1093/ps/82.3.347 12705392

[23] Zou ML Chen ZH Teng YY Liu SY Jia Y Zhang KW. The Smad dependent TGF-beta and BMP signaling pathway in bone remodeling and therapies. Front Mol Biosci. (2021) 8:593310. doi: 10.3389/fmolb.2021.593310 34026818 PMC8131681

